# The British Journals: Pathology, Practical Medicine, and Therapeutics

**Published:** 1841-07

**Authors:** 


*Edinburgh Medical and Surgical Journal. April,* 1841.

### PATHOLOGY, PRACTICAL MEDICINE, AND THERAPEUTICS.

### *Jaundice from Non-Elimination, together with remarks on the Pathological Condition, and Chemical Nature of the Bile.* By W. H. Lowe, m.d.

Dr. Lowe rejects the doctrine as purely hypothetical, that when jaundice appears suddenly and without premonitory symptoms, it is due to spasm, or to the opposite condition, paralysis of the gall-ducts. Jaundice from non-elimination may take place, either with the liver in a state of disease, or when there is no appreciable disease in that organ. Of the latter species, Dr. Lowe furnishes a single case of his own, and refers to others. According to Le Canu, three views are entertained by chemists of the condition of the blood of icteric patients. Some believe that it always contains bile: others, that it contains none, but only a particular colouring matter: others adopt a middle view, and think that the blood of such patients, without containing all the constituents of bile, holds its colouring principles.

Dr. Lowe, without attempting to offer an exact analysis of the bile, gives the result of some experiments of his own, "which have been conducted on a somewhat extensive scale." He endeavours to show that the resin of Gmelin, the picromel of Thenard, and the choleic and choloid acids of Demarcay, are "one and the same thing." We must refer our readers to the author's paper, for the experiments on which he grounds this view.

*Edinburgh Medical and Surgical Journal. April,* 1841.

---

### *On the Diagnosis of Abdominal Inflammations.* By William Griffin, m.d, m.r.c.s.e., Physician to the County of Limerick Infirmary, &c.

[This paper contains some new and striking illustrations of the important practical fact to which Dr. Griffin had previously called attention, viz. that inflammatory, or other affections of the spinal cord, or of its nerves, at their origin, more frequently simulate abdominal and thoracic inflammations, and are more frequently mistaken and maltreated, than is at at all imagined.

We have not room to give the cases, but extract the greater part of the general observations.]

There are three effects very common to inflammation, or disorder in the spinal cord, or at the trunks of its nerves.: 1st, Superficial tenderness, more or less exquisite, and either limited to the integument immediately over or about the affected portion of the cord, or extending thence to the front of the abdomen or thorax, in the direction of the spinal nerves; or occupying the whole cutaneous surface of those parts of the body, which are below the portion of the cord affected. 2dly, Pain either close to the affected portion of the cord, or at the extremities of the nerves, which have their origin there; or in the ganglionic nerves supplying the viscera, which have connexion with that portion of the cord. 3dly, Loss of power evinced in partial or complete palsy of the parts or organs, to which the affected nerves are distributed. These effects often occur simultaneously, but any one of them may also occur independently of the existence of the others, offering very strong evidence that the sensibility of the surface or skin and that which exists in internal organs is dependent upon nerves, which, though sentient, are as distinct from one another as they are from those on which the power of motion depends. Keeping these ordinary effects of disorder of the spinal cord in view, it must appear obvious, when we detect soreness or tenderness on pressure in the region of the liver or spleen, or of the lower abdominal viscera, how important and essential it is for us to ascertain whether the soreness be superficial or deep-seated, which we can almost always do by careful examination. Again, when pain is complained of in the region of the liver, or middle or lower parts of the abdomen, how necessary is it to ascertain, whether, as in the case of soreness, it be superficial; and, if deep-seated, whether it be merely an affection of the nerves of the part, and probably connected with some affection of the adjoining portion of the spinal cord, or whether the internal organ itself be in a state of acute or chronic inflammation. Finally, if there be oppression in breathing, whether it arises from deficient action in the respiratory nerves, and, as a consequence, imperfect action of the respiratory muscles, or imperfect performance of the process of oxygenation of the blood in the lungs; or from actual inflammation, or organic disease of the mucous membrane, or parenchyma of these organs. Or if there be obstinate constipation of bowels, whether it depends on spasm, or enteric inflammation; or whether purely or deficient power, or partial palsy of the muscular fibres of the intestines.

In determining the diagnosis of abdominal inflammation, where both pain and tenderness on pressure exist, we should always endeavour to ascertain:

1st. Whether there be any pain or tenderness on pressure in the corresponding portion of the spinal column; because, if there be, although it may not absolutely decide whether inflammation be present or not, it is quite sufficient to account for both the pain and tenderness, without assuming the existence of any inflammation.

2d. Whether there be no spinal tenderness or pain, the soreness of the abdomen be superficial or deep-seated, which may be ascertained with tolerable certainty in all cases by an examination directed to that end. And whether, if both superficial and deep-seated, as it usually is, in peritoneal inflammation, gentle, steady pressure with the flat of the hand can be easier borne, than with the points of the fingers. In pain and soreness from affection of the spinal nerves it commonly can be so borne, while in peritonitis every kind of pressure, and even the weight of the bed-clothes, is very distressing.

3d. Whether the boundaries of the pain or soreness extend beyond what the suspected inflammation could produce. Thus, if inflammation of the liver be suspected, and we find the soreness extending to the spine of the ilium, or groin, or to the opposite side of the abdomen to which the liver does not extend, it is obvious, the soreness cannot be attributable to mere disease of that organ. Again, if the whole abdomen be tender to the touch in a case otherwise closely resembling peritonitis, and we find the tenderness is not confined to the abdomen, but extends over the hips and lower extremities, it is obvious, we can attach no importance to the abdominal soreness as a sign of inflammation.

Finally, it should be recollected that constipation may depend on mere loss of power in the intestinal nerves, as well as on spasm, obstruction, or inflammation, as the treatment in each case must necessarily be modified or directed by the supposed cause of this symptom. *Dublin Journal. May,* 1841.

---

*Observations on Dropsy of the Pericardium.* By JOHN MACKENZIE, Esq., Surgeon.

THE diagnosis of hydrops pericardii is still involved in obscurity and uncertainty. The difficulty connected with this subject arises from the rare occurrence of the disease, unassociated with effusion into the pleural cavity, and to the still rarer opportunities of verifying such cases as do occur, by post-mortem examination. Having for several years been attached to a large military hospital in Russia, where chronic inflammation of the pericardium and consequent effusion were frequently met with, and where every facility of examining the bodies of those who died was afforded, I observed that, in those cases where there was co-existing effusion into the pleural cavity, the patients, from the commencement of the disease, could not bear the horizontal posture; and that, where the effusion was confined to the pericardium the patients preferred to be with the head remarkably low, till general effusion took place, and the breathing became laborious: of these, some preferred to sleep on their face, inclining to the left side, in such a manner as to make the region of the heart the most depending part, whilst others lay on their back. After death, I found in some of these cases that the pericardium occupied nearly the whole of the chest, and contained from six to ten pounds of fluid. In two cases there was not a vestige of the left lung to be found, except a thin layer of cellular substance.

The reason why the horizontal posture was preferred in these cases, appears to be, that the weight of the pericardium was taken off the diaphragm and rested on the spine or the ribs. It is to be observed, that in hydrothorax, when the patient lies with his head low, the water in the chest flows back and presses on the root of the lungs, preventing a free ingress of air, and thereby causing dyspnœa and sense of suffocation; and that in hydrops pericardii this cannot take place, in consequence of the water being inclosed in a firm bag which is bound down in its place by its attachment to the diaphragm. A very singular case of this disease came under my observation, where the patient could lie in all postures equally well, and experienced no inconvenience whatever; yet after death, which was caused by a sudden attack of inflammation of the right lung, the pericardium was found to occupy the whole of the left side of the chest, from the clavicle to the diaphragm, and contained ten pounds of thick brown fluid. *Lancet. April* 17, 1841.

---

*New method of employing Iodine in Phthisis.* By A. Leigh, m.b., Jersey.

I direct the patient to apply a sufficient quantity of iodine ointment on the ribs, under both axillæ, and to cover the head with the bed-clothes, to breathe the iodine volatilized by the heat of the axillæ; the ointment produces counter-irritation on the skin where it is applied, and is to be repeated according to circumstances. This method has appeared to me to arrest the progress of the disease. *London Medical Gazette. May* 28, 1841.

